# Genetic Improvement and Functional Characterization of AAP1 Gene for Enhancing Nitrogen Use Efficiency in Maize

**DOI:** 10.3390/plants14142242

**Published:** 2025-07-21

**Authors:** Mo Zhu, Ziyu Wang, Shijie Li, Siping Han

**Affiliations:** Institute of Agricultural Biotechnology, Jilin Academy of Agricultural Sciences (Northeast Agricultural Research Center of China), Changchun 130033, China; zhumo8989@163.com (M.Z.); wziyu86@163.com (Z.W.); 13278524455@163.com (S.L.)

**Keywords:** NUE, AAPs, maize, phylogenetic analysis, expression analysis

## Abstract

Nitrogen use efficiency remains the primary bottleneck for sustainable maize production. This study elucidates the functional mechanisms of the amino acid transporter *ZmAAP1* in nitrogen absorption and stress resilience. Through systematic evolutionary analysis of 55 maize inbred lines, we discovered that the *ZmAAP1* gene family exhibits distinct chromosomal localization (Chr7 and Chr9) and functional domain diversification (e.g., group 10-specific motifs 11/12), indicating species-specific adaptive evolution. Integrative analysis of promoter cis-elements and multi-omics data confirmed the root-preferential expression of ZmAAP1 under drought stress, mediated via the ABA-DRE signaling pathway. To validate its biological role, we generated transgenic maize lines expressing Arabidopsis thaliana *AtAAP1* via Agrobacterium-mediated transformation. Three generations of genetic stability screening confirmed the stable genomic integration and root-specific accumulation of the AtAAP1 protein (Southern blot/Western blot). Field trials demonstrated that low-N conditions enhanced the following transgenic traits: the chlorophyll content increased by 13.5%, and the aboveground biomass improved by 7.2%. Under high-N regimes, the gene-pyramided hybrid ZD958 (AAP1 + AAP1) achieved a 12.3% yield advantage over conventional varieties. Our findings reveal *ZmAAP1*’s dual role in root development and long-distance nitrogen transport, establishing it as a pivotal target for molecular breeding. This study provides actionable genetic resources for enhancing NUE in maize production systems.

## 1. Introduction

Nitrogen (N) is a fundamental component of plant growth and development and critically mediates adaptive responses to biotic and abiotic stresses [1,2,3]. While the “Green Revolution” brought remarkable yield increases through the widespread use of nitrogen fertilizers [4], a critical issue persists: crops absorb and utilize less than half of the nitrogen applied [5,6,7]. N losses caused by liquefaction, evacuation, and denitrification exacerbate environmental pollution and threaten ecosystem sustainability and human health [8,9,10,11]. Global population growth, expected to reach 9 billion people by 2050, will require an increase in agricultural productivity of 70 to 100 percent [12,13], making improving Nitrogen use efficiency (NUE) a scientific and socio-economic necessity [14,15].

Nitrogen use efficiency (NUE) is a polygenic feature modulated by genetic architecture and environmental interactions [3,16,17]. Plants rely on a diverse set of transporters to absorb nitrogen from the soil, each with unique substrate specificities and affinities [18,19,20,21]. Among these transports, amino acid transporters (AATs) stand out for their role in the uptake, translocation, and redistribution of amino acids, the primary form of organic nitrogen [22,23]. The amino acid permease (AAP) family, particularly *AAP1*, has attracted attention for its involvement in ammoniacal nitrogen metabolism, especially in phloem loading and seed development [24,25,26]. One of the well-studied proteins in *Arabidopsis*, *AAP1*, is known to facilitate amino acid import into embryos, directly influencing seed protein content and nitrogen use efficiency [27,28]. The overexpression of *AAP1* in legumes has been shown to enhance nitrogen uptake into embryos and boost seed yields under high-nitrogen conditions [29,30,31]. Similarly, in rice, *OsAAP1* regulates fertility and grain development by modulating the transport of N [32,33]. These findings identify AAP1 orthologs as promising targets for enhancing the nitrogen use efficiency (NUE) of cultures.

Despite advances in model species, the function of *Zea mays ZmAAP1* remains poorly characterized. Critical knowledge gaps exist regarding its regulatory role in the development of the stem architecture, the division of amino acids, and the transcriptional responses to the variable availability of N. The molecular mechanisms that regulate *ZmAAP1*-mediated N transport, in particular its interactions with other N transporters and metabolic networks, need to be clarified. Moreover, the potential of the genetic manipulation of *ZmAAP1* to improve NUE and stress resistance (drought, thermal extremes) under N-suboptimal conditions remains unvalidated. Closing these knowledge gaps is crucial for developing organic corn varieties.

This study examines the functional role of *ZmAAP1* in the N-economy of corn through bioinformatics, molecular, physiological, and integrated field approaches. We also analyze sequence structure changes during its evolutionary process and explore its possible biological functions, particularly in root development and amino acid allocation. The expression patterns of *AAP1* are characterized under different nitrogen regimes, including high, medium, and low nitrogen levels, as well as under abiotic stress conditions such as drought and heat. The molecular mechanisms underlying *AAP1*-mediated nitrogen transport are elucidated, with a focus on its interactions with other nitrogen transporters and metabolic pathways. Additionally, the potential of *AAP1* overexpression to improve NUE and yield in maize will be evaluated, particularly in transgenic lines engineered for enhanced amino acid absorption and transport efficiency. We posit that *ZmAAP1* acts as a central regulator coordinating N allocation and metabolic integration, with the translational potential to enhance NUE through targeted genetic improvement. This study will elucidate molecular mechanisms underlying *Zea mays* N assimilation, identifying candidate targets for sustainable intensification. By resolving *ZmAAP1*’s regulatory role in N transport coordination and stress adaptation, our findings will advance genetic strategies to optimize NUE under fluctuating N availability and climate extremes. Translational outcomes include developing maize varieties with enhanced N-use efficiency, addressing critical gaps in global food security while mitigating fertilizer overuse and environmental degradation.

## 2. Results

### 2.1. Identification of ZmAAP1 Genes in 55 Maize Inbred Lines

The *ZmAAP1* genes were identified in 55 endogenous maize inbred lines using BLAST analyses and a hidden Markov model (HMM) with *AtAAP1* from *Arabidopsis* as a reference. Each suspected sequence of *ZmAAP1* was validated using CDD and Pfam databases. Detailed information on the gene ID, the location of the chromosome, the number of exons, the length of the proteins, the molecular weight, the isoelectric point, and the subcellular location is provided in Table 1.

Most *ZmAAP1* genes are localized on Chr7, with exceptions such as CML103 and PH207 on Chr9 or frame 7.87. The length of the proteins varies from 475 (MEX) to 512 (B104) amino acids, with molecular weights between 51358.6 Da (MEX) and 55367.24 Da (B104). The isoelectric points range from 7.2 (SK) to 8.42 (CML103). The predicted subcellular locations include the plasma membrane, the endoplasmic reticulum, the cytoplasm, the chloroplast, the peroxisome, and the Golgi device, with some proteins having multiple locations.

These results demonstrate the genetic diversity of *ZmAAP1* across endogenous corn lines. Although *ZmAAP1* shares a high homology with *Arabidopsis AAP1*, its structural and functional variations suggest potential roles in nitrogen transport, stress responses, and developmental regulation. Other studies on *ZmAAP1* could provide information on improving the effectiveness of nitrogen use in corn.

### 2.2. Cluster Analysis, Sequence Motif, and Conserved Domain Analysis of AAP1 Proteins

**Figure 1:**
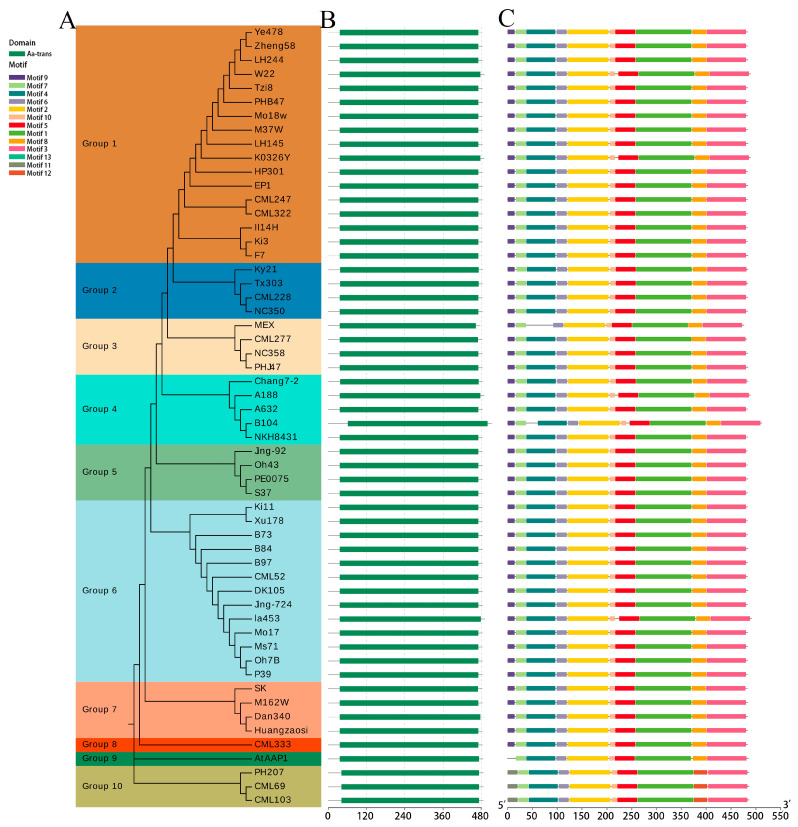
Evolutionary tree, domain, and motif analysis of *AAP1* genes in 55 maize inbred lines and *Arabidopsis*. Note: (**A**) Phylogenetic tree of AAP1 proteins, with distinct subgroups highlighted by different colors. (**B**) Protein domains of AAP1s, indicated in dark green. (**C**) Motif compositions of AAP1s, with different colors representing various motifs; the ruler at the bottom indicates motif length.

To investigate the evolutionary relationships between the ZmAAP1 proteins, a phylogenetic tree was constructed using amino acid sequences from *Arabidopsis thaliana* and corn (Figure 1). The ZmAAP1 family was divided into ten groups (group 1 to group 10), with group 8 containing AtAAP1 from Arabidopsis thaliana. Groups 8 and 10 form different branches that indicate distant phylogenetic relationships. Notably, the three members of group 10 are located on chromosome 9, indicating functional similarities that differ from those of the other groups.

Structural analysis found that the pattern distribution correlated with the phylogenetic classification. The proteins of the same group share similar patterns, while variations in the compositions of the patterns may reflect functional diversification. All ZmAAP1 proteins contain the preserved Aa-trans domain, but significant heterogeneity lies in the composition of the motif. Groups 9 and 10 are missing from motif 9, a feature that is also missing in AtAAP1. Group 10 has only motifs 11 and 12, which represent motifs 9 and 12, respectively. They were replaced by motif 8.

These structural variations show the evolutionary divergence of ZmAAP1 proteins. The resulting Aa-trans domain indicates a common functional core, while the pattern differences indicate a potential functional specialization. For example, the unique motifs of group 10 may confer specific biochemical properties. These results provide insights into the structural and functional diversity of AAP1 proteins in corn with implications for improving the effectiveness of nitrogen use and stress tolerance in crops.

### 2.3. Cis-Element Analysis of the AAP1 Genes

**Figure 2:**
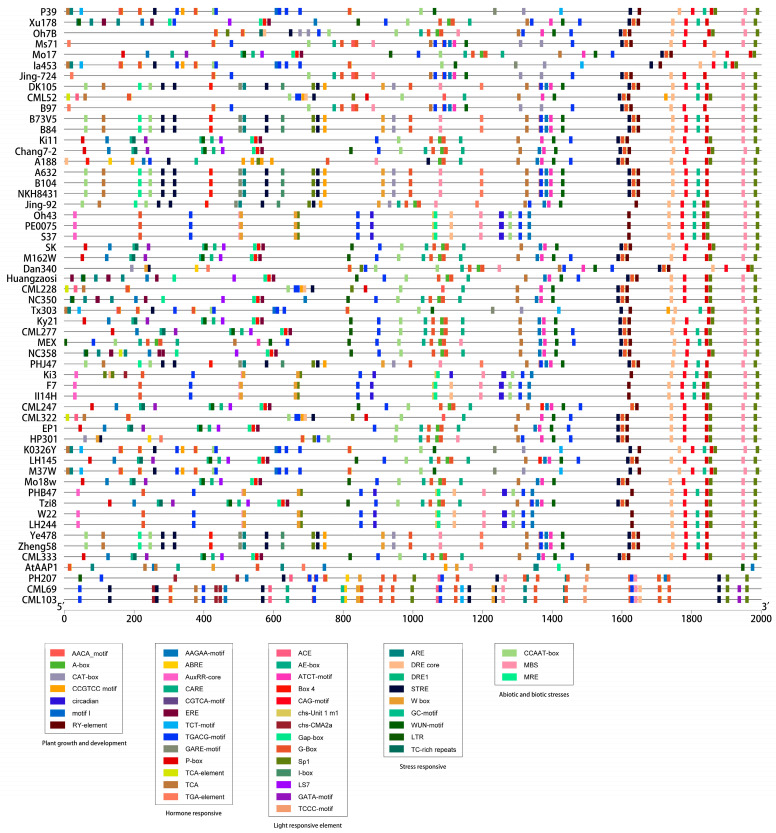
Analysis of cis-acting elements in upstream promoter regions of AAP1s. Different cis-acting elements are represented with different color blocks.

Analyzing cis elements in the promoter regions of the *ZmAAP1* genes is crucial for understanding their regulatory mechanisms and functional roles. The sequences prior to 2000 bp for the genes *ZmAAP1* of various maize lines (Figure 2) and *AtAAP1* of *Arabidopsis thaliana* were analyzed using the PlantCARE database.

The results show that the *ZmAAP1* promoters contain several cis elements associated with hormone response, stress response, and light response. Notably, light-sensitive elements (e.g., G-box, GT1 pattern) and hormone-sensitive elements (e.g., ABRE) are very abundant, suggesting that the expression of *ZmAAP1* is regulated by environmental and developmental signals. In addition, stress-sensitive elements such as DRE point to a potential role for *ZmAAP1* in adapting to abiotic stress.

These results provide information about the regulatory network that controls *ZmAAP1* expression and its potential involvement in stress responses and nitrogen metabolism. Further studies are needed to investigate how these cis elements influence *ZmAAP1* function and develop strategies to improve the effectiveness of nitrogen use and stress tolerance in corn.

### 2.4. Expression Profiles of ZmAAP1 in Maize During Growth and Development

**Figure 3:**
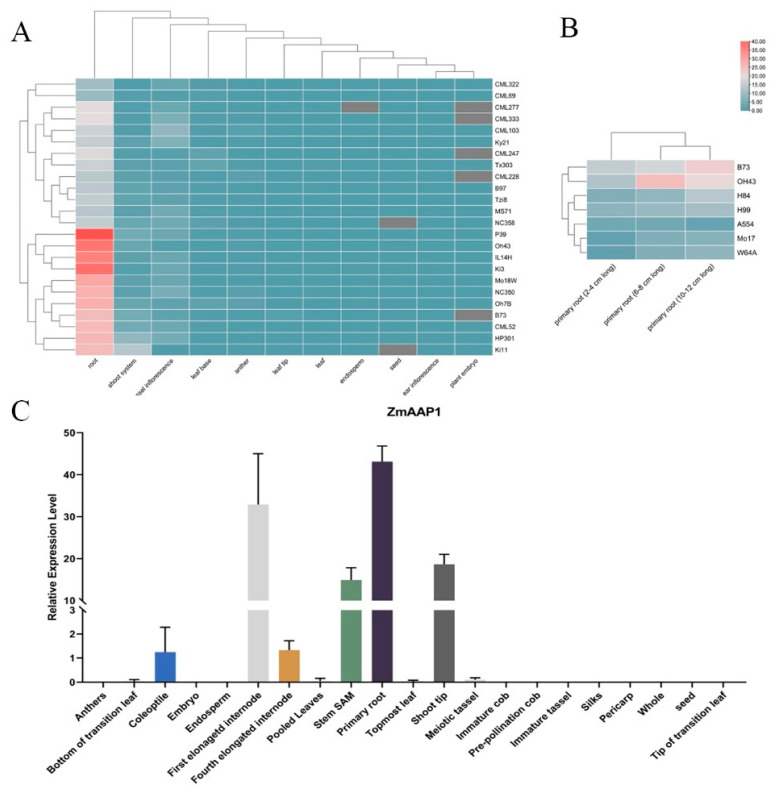
The multi-dimensional expression profiling of the *ZmAAP1* gene in maize inbred lines. Note: (**A**) Expression profiles of ZmAAP1 genes in different maize inbred lines across various organs (root, shoot system, tassel inflorescence, leaf base, anther, leaf tip, leaf, endosperm, seed, ear inflorescence, and plant embryo). The color gradient from blue to red represents low to high expression levels of ZmAAP1. (**B**) Expression profiles of ZmAAP1 genes in different root systems. Differential expression of ZmAAP1s in primary root (2–4 cm), primary root (6–8 cm), and primary root (10–12 cm). (**C**) The expression of ZmAAP1 in different organs. Data in this figure are the mean ± SD. Statistical differences are analyzed using a two-tailed unpaired *t* test with Welch’s correction.

RNA-seq data from 24 maize inbred lines were analyzed to study the pattern of *ZmAAP1* expression during growth and development (Figure 3A). *ZmAAP1* showed tissue-specific and inbred line-specific expression, with the highest levels constantly observed in the roots. Breeding lines such as P39, Oh43, IL14H, and Ki3 showed particularly high tribal expression, while some lines also showed significant expression in purification systems and butterfly flowers.

In the B73 inbred line, *ZmAAP1* was strongly expressed in five key tissues: the coleoptile, the first prolonged internode, the SAM of the back, the primary root, and the thrust tip (Figure 3B). This constitutive expression pattern suggests a critical role for *ZmAAP1* in the growth and development of these tissues. High expression in the roots addresses its potential function in nitrogen absorption and assimilation, while expression in the spinel tips and branch SAM indicates participation in apical growth and merista maintenance. This implies that the *ZmAAP1* genes may play a central role in the growth and development of organs, particularly in the roots and primary stem.

RNA-seq analysis was performed to examine *ZmAAP1* expression in three zones (2–4 cm, 6–8 cm, and 10–12 cm) of the primary root on seven lines of corn (Figure 3C). In B73, *ZmAAP1* expression peaked in the 10–12 cm range, indicating a role in root maturation or nutrient transport. H84 and H99 showed similar patterns, while Oh43 showed the highest expression in the 6 to 8 cm area. These results show that *ZmAAP1* expression is spatially regulated and varies from line to line, reflecting potential adaptations to environmental conditions or development.

### 2.5. Expression Patterns of ZmAAP1 Under Heat and Drought Stress

**Figure 4:**
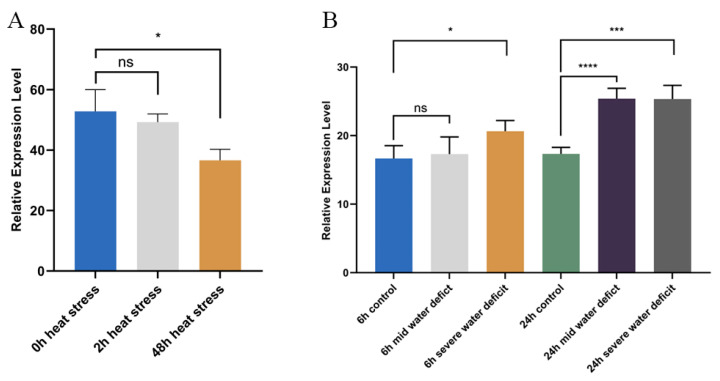
Abiotic stress-induced expression profiling of ZmAAP1 via qRT-PCR. Note: (**A**) Heat stress response and quantitative analysis of ZmAAP1 expression in maize seedlings under heat stress (35 °C, 6 h). Data represent mean ± SD from three independent biological replicates. Statistical significance (*) indicates differential expression relative to the control (25 °C) (* *p* < 0.05, *p* < 0.01; ns = >0.1; Welch’s *t*-test). (**B**) Drought stress response and temporal expression dynamics of ZmAAP1 during progressive drought treatment (10%, 15%, 20% PEG6000 simulation). Data are expressed as mean ± SD (n = 3). Asterisks denote significant upregulation compared to the baseline (0% PEG) (**** *p* < 0.0001, *** *p*< 0.003, * *p* < 0.05; ns = >0.1; Welch’s *t*-test).

Due to the presence of cis-active elements in relation to abiotic stress responses in the *ZmAAP1* promoter, the expression pattern of *ZmAAP1* under thermal and dry stress was analyzed to study its role in stress adaptation (Figure 4). Under high temperature stress, *ZmAAP1* expression in B73 decreased gradually, with a significant reduction after 48 h. Under drought stress, however, *ZmAAP1* expression initially increased after 6 h and showed a significant increase after 24 h. These results suggest that *ZmAAP1* is differentially regulated under the stresses of heat and drought, potentially contributing to stress adaptation mechanisms.

### 2.6. Analysis of the Insertion Sequence Stability of the Transgenic Maize Lines na1, na2, and na3 Across Generations

The amino acid carrier gene *AtAAP1* of *Arabidopsis thaliana* was cloned into the vector pCAM-UPN, which also contains the genetic marker strain selectively, resulting in the recombinant vector pCAM-UPN::*AtAAP1*. This vector was introduced via agrobacterium-mediated transformation into the embryonic tissue of the inbred line of corn Zheng58. The positive transgenic plants were selected via herbicide and PCR detection, identifying three high-performance events (na1, na2 and na3) depending on amino acid absorption and transport efficiency that were inherited stably over several generations (Figures S1 and S2). Specific PCRs (PR1 and PR2) were designed to detect the transgene in generations T4-T6 and confirm stable integration and inheritance (Figure S3).

**Figure 5:**
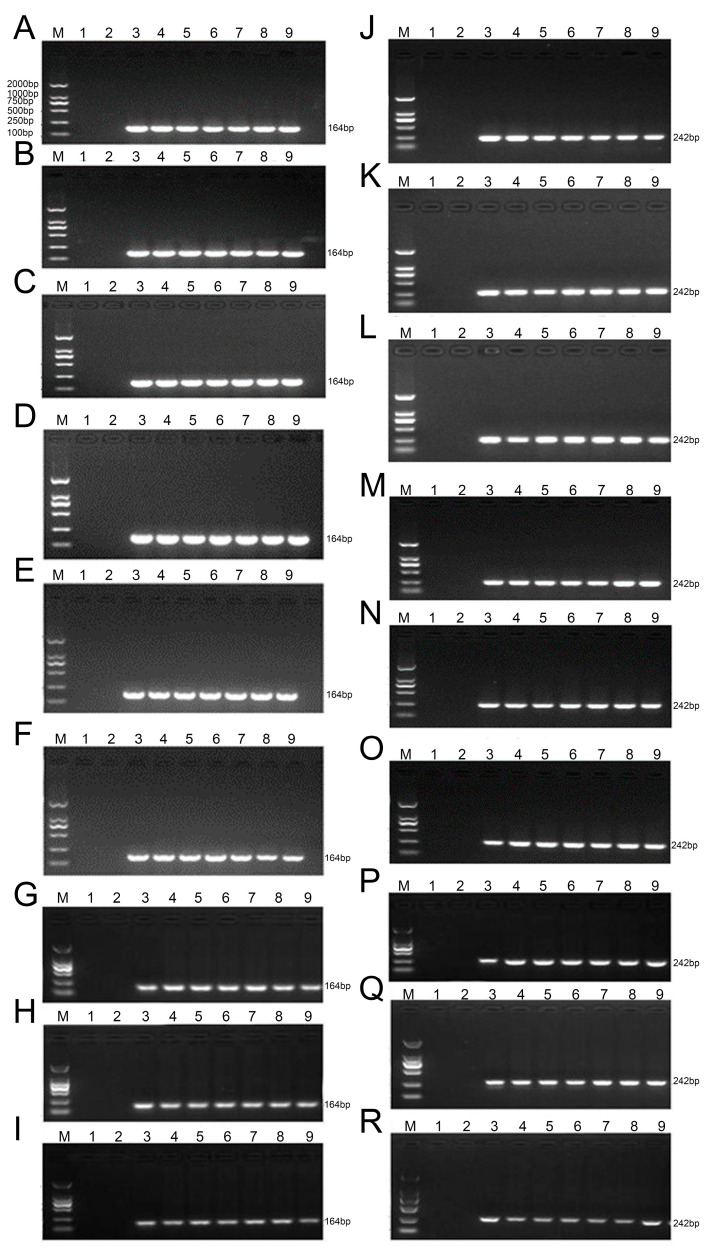
RT-PCR analysis of the *AtAAP1* gene in different organs of na1, na2, and na3 corn across three consecutive generations. Note: M: DL 2000 marker; 1: water; 2: negative control (HiII); 3: positive control (pCAM-UPN::*AtAAP1* plasmid); 4–9: root, stem, leaf, silk, tassel, and kernel; (**A**–**I**) AtAAP, (**J**–**R**) bar; (**A**–**C**,**J**–**L**) T4–T6 generations of na1, (**D**–**F**,**M**–**O**) T4–T6 generations of na2, (**G**–**I**,**P**–**R**) T4–T6 generations of na3.

**Figure 6:**
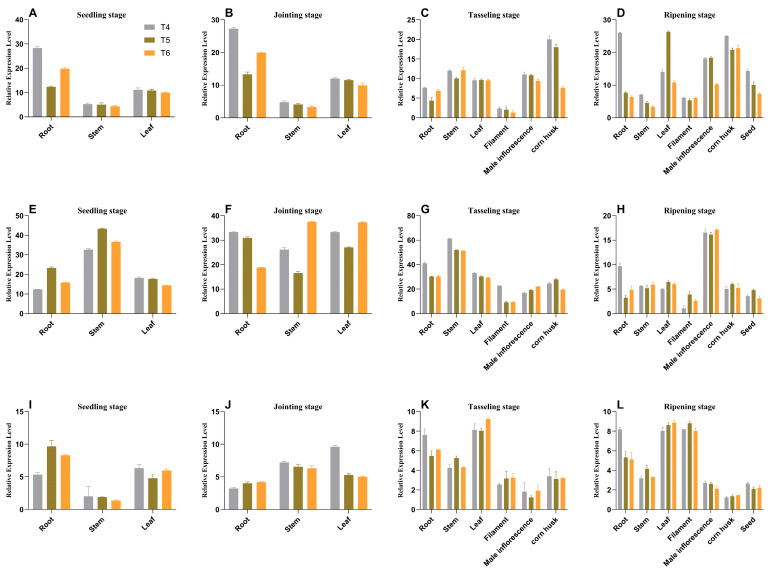
Analysis of temporal and spatial expression patterns of the *AtAAP1* gene in the transgenic maize na1, na2, and na3. Note: Data are presented as mean ± SD, with n = 3; subplots (**A**–**D**) correspond to na1 genotype maize, (**E**–**H**) to na2 genotype maize, and (**I**–**L**) to na3 genotype maize. Developmental stages (distinguished by rows within each group, 4 stages total): (**A**,**E**,**I**)—seedling stage; (**B**,**F**,**J**)—joint stage; (**C**,**G**,**K**)—tasseling stage; (**D**,**H**,**L**)—ripening stage. Color coding and genotype/treatment labels: bar chart colors (3 bars per subplot): gray (T4)—represents the na1 genotype (corresponding to subplots (**A**,**E**,**I**)); green (T5)—represents the na2 genotype (corresponding to subplots (**B**,**F**,**J**)); orange (T6)—represents the na3 genotype (corresponding to subplots (**C**,**G**,**K**)).

**Figure 7:**
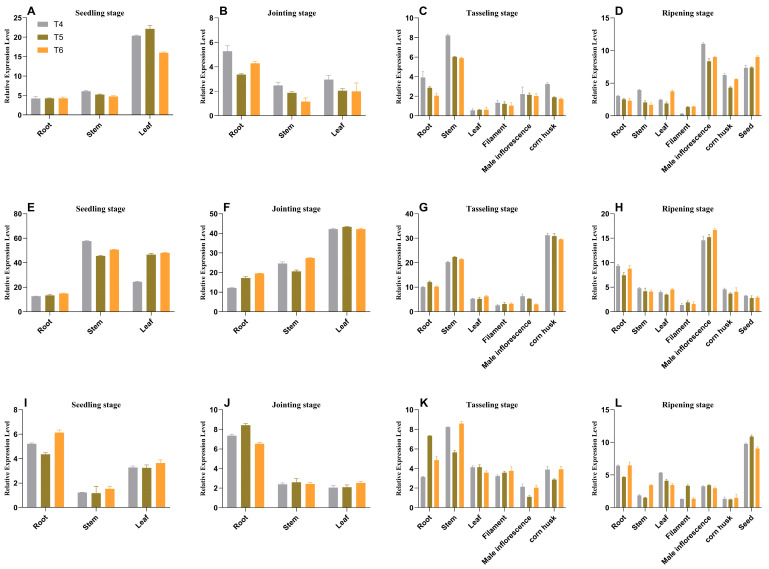
Analysis of temporal and spatial expression patterns of the *bar* gene in transgenic maize na1, na2, and na3. Note: Data are presented as mean ± SD, with n = 3; subplots (**A**–**D**) correspond to na1 genotype maize, (**E**–**H**) to na2 genotype maize, and (**I**–**L**) to na3 genotype maize. Developmental stages (distinguished by rows within each group, 4 stages total): (**A**,**E**,**I**)—seedling stage; (**B**,**F**,**J**)—joint stage; (**C**,**G**,**K**) tasseling stage; (**D**,**H**,**L**) ripening stage. Color coding and genotype/treatment labels: bar chart colors (3 bars per subplot): gray (T4)—represents the na1 genotype (corresponding to subplots (**A**,**E**,**I**)); green (T5)—represents the na2 genotype (corresponding to subplots (**B**,**F**,**J**)); orange (T6): represents the na3 genotype (corresponding to subplots (**C**,**G**,**K**)).

Analysis of expression using SqRT-PCR and qRT-PCR revealed that the *AtAAP1* and *bar* genes were expressed in different tissues (root, stem, leaf, silk, bag, grain, and shell) through different stages of development (plant, joint, bag, and maturity) (Figure 5, Figure 6 and Figure 7). In na1, *AtAAP1* expression was highest in the roots during the early stages and in the shells during tasseling, while *bar* expression peaked in the leaves and roots at various stages. Similar patterns were observed in na2 and na3, with some variations in tissue-specific expression levels.

**Figure 8:**
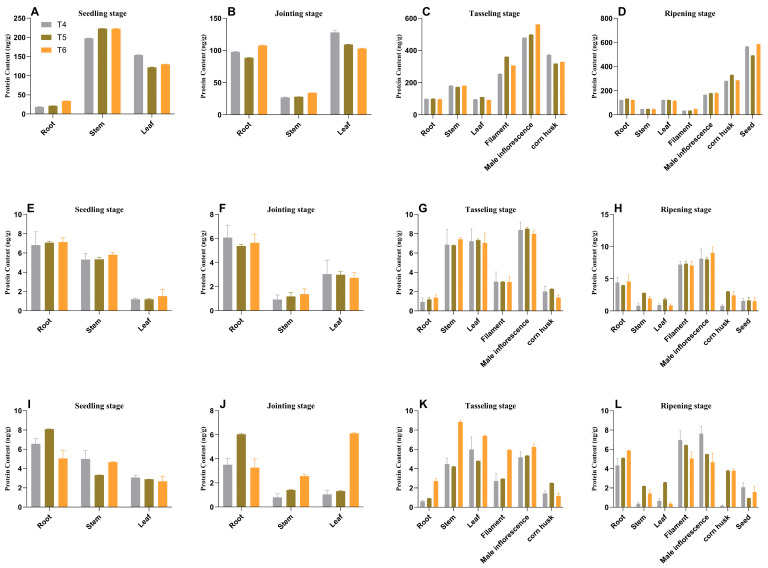
Content of AtAAP1 protein in different organs of transgenic maize. Note: Data are presented as mean ± SD, with n = 3; subplots (**A**–**D**) correspond to na1 genotype maize, (**E**–**H**) to na2 genotype maize, and (**I**–**L**) to na3 genotype maize. Developmental stages (distinguished by rows within each group, 4 stages total): (**A**,**E**,**I**) seedling stage; (**B**,**F**,**J**) joint stage; (**C**,**G**,**K**) tasseling stage; (**D**,**H**,**L**) ripening stage. Color coding and genotype/treatment labels: bar chart colors (3 bars per subplot): gray (T4)—represents the na1 genotype (corresponding to subplots (**A**,**E**,**I**)); green (T5)—represents the na2 genotype (corresponding to subplots (**B**,**F**,**J**)); orange (T6)—represents the na3 genotype (corresponding to subplots (**C**,**G**,**K**)).

**Figure 9:**
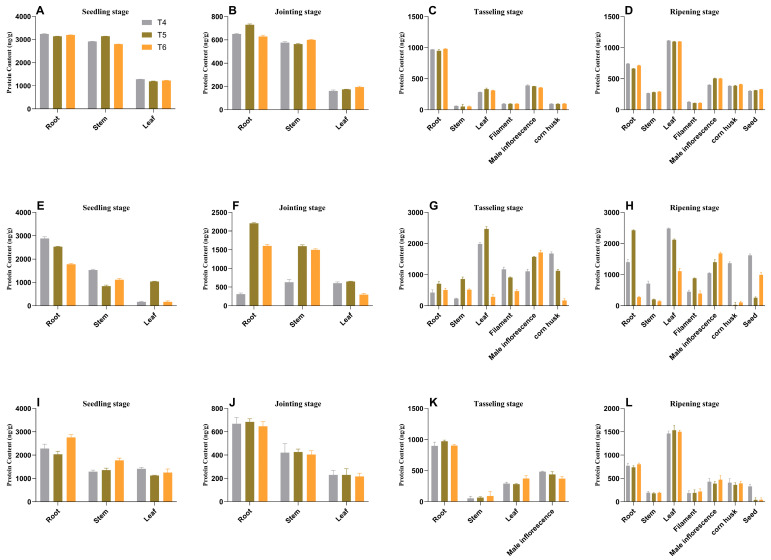
Content of the PAT protein in different organs of transgenic maize across generations (ng/g). Note: Data are presented as mean ± SD, with n = 3; subplots (**A**–**D**) correspond to na1 genotype maize, (**E**–**H**) to na2 genotype maize, and (**I**–**L**) to na3 genotype maize. Developmental stages (distinguished by rows within each group, 4 stages total): (**A**,**E**,**I**) seedling stage; (**B**,**F**,**J**) joint stage; (**C**,**G**,**K**) tasseling stage; (**D**,**H**,**L**) ripening stage. Color coding and genotype/treatment labels: bar chart colors (3 bars per subplot): gray (T4)—represents the na1 genotype (corresponding to subplots (**A**,**E**,**I**)); green (T5)—represents the na2 genotype (corresponding to subplots (**B**,**F**,**J**)); orange (T6)—represents the na3 genotype (corresponding to subplots (**C**,**G**,**K**)).

ELISA analysis confirmed the presence of AtAAP1 and PAT proteins in all the tissues studied, with the expression levels remaining stable over generations (Figure 8 and Figure 9). For example, in na1, the AtAAP1 protein levels were highest in the stems during sowing, in the leaves during the joint stage, and in the shells during cupping. The PAT protein levels were consistently high in the roots and leaves throughout the development stages.

These results demonstrate the integration and stable expression of the *AtAAP1* and *bar* genes in the transgenic lines of maize na1, na2, and na3. The consistent expression patterns across generations and tissues suggest that these lines are suitable for other studies on the effectiveness of nitrogen use and stress tolerance, with potential applications in improving crops.

### 2.7. Phenotypic Characterization of the Target Gene AtAAP1 in Transgenic Maize Lines

**Figure 10:**
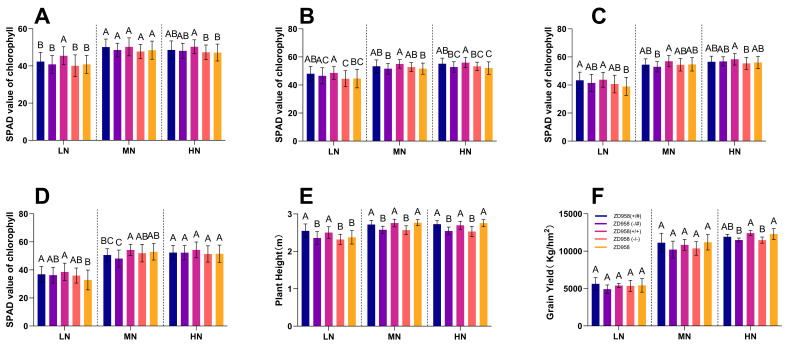
Phenotypic data of transgenic maize under different nitrogen conditions: (**A**) chlorophyll SPAD value at the seedling stage; (**B**) chlorophyll SPAD value at the joint stage; (**C**) chlorophyll SPAD value at the tasseling stage; (**D**) chlorophyll SPAD value at the maturity stage; (**E**) plant height; (**F**) maize yield. Note: Data in panels (**A**–**E**) represent the mean ± standard deviation of 10 biological replicates. Data in panel (**F**) represent yield (at 14% moisture) ± standard deviation. The yield measurement plot area was 18 m^2^. LN: no nitrogen fertilizer; MN: 70% nitrogen fertilizer; HN: 100% nitrogen fertilizer. Statistical difference (no significant difference for the same letter, significant difference for different letters, *p* < 0.05). In the seedling stage under LN conditions, the transgenic line ZD958 (+/#) showed a chlorophyll SPAD value 3.6% higher than the ZD958 (−/#) command. The pyramidal genetic hybrid ZD958 (+/+) showed a more pronounced advantage, with a 13.5% increase in chlorophyll content compared to ZD958 (−/−).

To clarify the phenotypic effects of *AtAAP1* in transgenic maize, an in-depth field study was performed during the intermediate phase of the test (Figure 10). A random block sign was used, with ten corn plants exhibiting comparable growth characteristics selected by each experimental field. Morphological and physiological characteristics, including plant height and chlorophyll SPAD values, were systematically evaluated via different nitrogen fertilization regimens. The measurements were carried out in the key stages of development, plants, compounds, touches, and maturation, allowing a detailed analysis of the dynamic changes induced by AtAAP1 throughout the plant’s growth cycle.

To assess the efficacy of the transgene in a hybrid genetic context, we hybridized the T6-generation AtAAP1 corn transformer with conventional inbred line Zheng 58 and Chang 7-2 cross lines and obtained positive, non-segregating homozyotic transgenic cross lines via continuous back-growth for seven generations and two self-growth stages. The transgenic genetic lines obtained were then used to bring together a transgenic hybrid and a genetically aggregated hybrid. These lines were later used to generate transgenic hybrids and hybrids in the gene pyramid, facilitating a comprehensive evaluation of transgene performance in complex genetic contexts. On-site assessments focused on the effectiveness of nutrient use, with detailed results summarized in Table 2.

In the jointing stage under MN conditions, ZD958 (+/#) maintained a 3.4% chlorophyllic advantage over ZD958 (−/#), while the ZD958 (+/+) hybrid showed a 4.3% increase over ZD958 (−/−).

In the tasseling stage under HN conditions, the chlorophyll content of ZD958 (+/#) was slightly lower (−0.6%) than that of ZD958 (−/#), possibly due to environmental variability. However, hybrid ZD958 (+/+) maintained a significant advantage of 5.3% over ZD958 (−/−) in terms of chlorophyll content.

In addition, we carried out a comparative analysis of plant height between different varieties of corn. Under LN conditions, ZD958 (+/#) showed an increase in plant height of about 7.2% over ZD958, while the plant height of the ZD958 (+/+) combination remained largely comparable to that of ZD958 (−/−). Under MN conditions, the plant height of ZD958 (+/#) was slightly lower than that of ZD958, while the combination of ZD958 (+/+) exceeded ZD958 (−/−) by about 7.4%. Under HN conditions, there was a minimal difference in plant height between ZD958 (+/#) and ZD958, but the combination ZD958 (+/+) maintained a significant difference of about 6.5% compared to ZD958 (−/−).

Nitrogen treatment performance has shown the following: The unique ZD958 (+/#) genetic hybrid showed the best biomass performance on the soil, highlighting its superior stability and adaptability in nitrogen-constrained environments. The hybrid in the ZD958 (+/+) gene pyramid produced less than ZD958 (+/#) but outperformed both ZD958 (−/#) and ZD958 (−/−), indicating better tolerance to low nitrogen content.

Under MN conditions, ZD958 (+/#) achieved high yields, reflecting the effective use of nitrogen. The ZD958 also performed well and came close to the performance of ZD958 (+/#). The ZD958 (+/+) hybrid showed improved performance but lagged behind ZD958 (+/#) and ZD958, perhaps due to a suboptimal adaptation to moderate nitrogen availability.

Under HN conditions, the ZD958 (+/+) hybrid achieved the best performance and demonstrated greater effectiveness in using nitrogen. ZD958 (+/#) and ZD958 followed closely and showed their high-performance potential under conditions with a high nitrogen content.

## 3. Discussion

The absorption of nitrogen and the effectiveness of nitrogen use in maize show significant genetic variations [34]. The overall efficiency of corn nitrogen use is likely to depend on several factors: the supply of nitrogen assimilates, their translocation, and their transformation during core formation. However, our knowledge of the coordination of these processes is incomplete. Recent studies show genetic differences in the long-distance transport of amino acids through phloema [32,35,36]. The concentration of amino acids and their effective movement in vascular tissue are key to determining grain yield [37]. In addition, AAP protein, an important carrier of amino acids, helps the movement of amino acids from plant organs to reproductive organs, affecting nitrogen efficiency and yield. While much research has focused on the absorption of inorganic nitrogen and the assimilation of ammonium in corn, less attention has been paid to the transport of amino acids. In this study, *ZmAAP1* was identified specifically among 55 lines of corn. An in-depth analysis of genetic and protein structures, their regulation of expression, and their phylogenetic properties was carried out. In addition, *AtAAP1* was successfully transferred into maize bath lines, and its phenotypic manifestations were accurately characterized via hybridization techniques and other molecular biological methods.

### 3.1. Genetic and Structural Diversity of ZmAAP1 Proteins in Maize

Identifying and characterizing the *ZmAAP1* genes on several innate maize lines revealed significant diversity in genomic localization, protein structure, and subcellular localization. This diversity suggests potential functional specialization during maize evolution, driven by mechanisms such as genetic recombination, mutation, and natural selection [14,15].

Phylogenetic analysis revealed different grouping patterns between the ZmAAP1 proteins, with groups 8 and 10 showing distant phylogenetic relationships. In particular, all members of group 10 are on chromosome 9, indicating a possible functional relationship that is different from the other groups. Structural analysis also highlighted the presence of conserved domains, such as the Aa-trans domain, in addition to variable domain, suggesting a central functionality between ZmAAP1 proteins with additional structural characteristics that confer unique properties. For example, the absence of pattern 9 in groups 9 and 10 and the unique presence of patterns 11 and 12 in group 10 can contribute to functional specialization.

The predicted subcellular locations of ZmAAP1 proteins, including the plasma membrane, endoplasmic network, and cytoplasm, suggest roles in membrane transport, protein movement, and intracellular signaling. The presence of ZmAAP1 in chloroplasts, perossosomes, and the Golgi apparatus also suggests participation in photosynthesis, perossosomal metabolism, and protein processing [22,23]. Future research should focus on the specific functions of the various ZmAAP1 proteins, their regulatory mechanisms, and their interaction networks, in particular with regard to nitrogen absorption and efficacy of use. This is crucial for harnessing the potential of ZmAAP1 proteins in efforts to improve crops.

### 3.2. Transcriptional Regulation and Functional Diversity of ZmAAP1

*ZmAAP1* expression profiles showed complex transcriptional regulation during corn growth and development. High levels of expression in roots, particularly in embedded lineages such as P39, Oh43, IL14H, and Ki3, indicate a critical role in root development and function. Constitutive expression in tissues such as the coleoptile, the SAM strain, and the thrust tip continues to support its participation in apical growth and meristemal care.

The spatial regulation of *ZmAAP1* expression across different root zones and embedded lines suggests a connection between transcriptional control and specific biological functions, such as root hair development, nutrient acquisition, or signal transduction. For example, high expression in the 10–12 cm area of B73 roots may indicate a role in root maturation or nutrient transport. In addition, the differential expression of *ZmAAP1* under heat and drought stress highlights its potential involvement in abiotic stress reactions. The progressive decrease in expression under high temperature stress and the significant increase under drought stress suggest that *ZmAAP1* may play a role in stress adaptation mechanisms.

### 3.3. Transgenic Maize Lines with Enhanced Amino Acid Transport Efficiency

The successful construction of transgenic maize lines (na1, na2 and na3) expressing *AtAAP1* showed the potential of genetic engineering to improve amino acid uptake and transport efficiency. Molecular characterization, including Southern blot and binding sequence analysis, confirmed the stable integration and inheritance of the transgene over several generations. Expression analysis revealed specific tissue and developmental patterns with relatively stable expression levels across generations, suggesting the robustness of the transgenic system.

*AtAAP1* significantly improved the efficiency of nitrogen use, chlorophyll content, and plant height and yield in transgenic corn. Under LN conditions, the monogenic hybrid ZD958 (*AAP1*) showed the highest biomass yield above ground and showed superior stability and adaptability. Under sufficient nitrogen conditions, the transgenic hybrid strain ZD958 (+/+) showed a yield advantage over the control (CK, ZD958), though the difference was not statistically significant. This may be attributed to the fact that AtAAP1 only enhances one physiological process in the nitrogen absorption and utilization pathway. In our unpublished studies, genes simultaneously enhancing nitrogen absorption, transport, and assimilation showed more direct and effective impacts on yield. Nevertheless, these results still confirm that AtAAP1 has the potential to save fertilizer and increase yields. This further validates the value and potential of gene pyramiding in breeding nutrient-efficient new varieties.

### 3.4. Implications for Crop Improvement

The *AAP1* gene family represents a valuable genetic resource for improving NUE, harvest resistance, and yield [38]. By integrating *AtAAP1* with other favorable genetic determinants through advanced genetic engineering techniques, it may be possible to develop “super harvests” with increased yields, superior qualitative characteristics, and increased stress tolerance [39,40]. In addition, advances in gene processing technologies offer the potential to precisely modulate *AtAAP1* expression, refine plant signs, and contribute to sustainable agriculture [41,42].

Finally, the family of the *AAP1* gene has enormous potential to improve the absorption and effective use of nitrogen, chlorophyll content, plant biomass, and grain yield. These results offer a promising opportunity to address global food security challenges and promote sustainable agricultural practices.

## 4. Methods

### 4.1. Identification and Sequence Analysis of the ZmAAP1 Genes in Maize

To enhance the precision of identification, a dual approach was employed to analyze the ZmAAP1s in various maize inbred lines. We used two methods to search for the target protein. Firstly, by searching the NCBI database (https://www.ncbi.nlm.nih.gov/ (accessed on 20 March 2024)) and BLAST (https://blast.ncbi.nlm.nih.gov/Blast.cgi (accessed on 20 March 2024)), we downloaded the entire genome file (V5 version) and gene sequence annotation file of maize ZmB73-REFERENCE NAM_5.0.55. Moreover, through TAIR (https://www.arabidopsis.org/ (accessed on 23 March 2024)) and NCBI (https://www.ncbi.nlm.nih.gov/ (accessed on 23 March 2024)), we obtained information on the Arabidopsis *AAP* gene family for comparison using nlm.nih.gov/ and used Arabidopsis AtAAP1 protein sequence alignment to extract homologous AAP proteins from the maize genome. Secondly, from the Pfam protein family domain database (http://pfam.xfam.org/ (accessed on 5 April 2024)), we obtained the conservative domain file containing PF01490.21 and all hidden Markov model files (Pfam-A), then used the corn proteome file and the “Simple HMM Search” function in TBtools Version 2.146 to search for AAP1 homologous proteins in corn. We performed bidirectional validation on the two results. All maize proteins underwent screening using this maize-specific model, with those with an e-value <0.01 being selected. To further validate the ZmAAP1 proteins, the Pfam and InterPro databases (http://www.ebi.ac.uk/interpro/ (accessed on 25 March 2024)) were utilized. Proteins confirmed through domain and database screenings were deemed to be ZmAAP1s. Corresponding CDS and protein sequences were subsequently extracted based on protein identification.

The predicted subcellular localization of AAP1s was analyzed using WoLF PSORTII (http://www.genscript.com/wolf-psort.html (accessed on 29 March 2024)).

### 4.2. Domain and Motif Structure Diagram of ZmAAP1s

The MEME [43] program (http://meme-suite.org/ (accessed on 11 April 2024)) was used to identify conserved motifs in the ZmAAP1 protein with the following parameters: any number of repetitions, a minimum of 6 patterns, a maximum of 50 patterns, an optimal size of 10–200 amino acids, and an expected e-value of less than 1 × 10^−48^. The structure of the ZmAAP1 proteins was visualized by comparing the coding sequences and genomic sequences using TBtools [44]. The isoelectric point and molecular weight of the ZmAAP1 protein were estimated using ExPASy (http://expasy.org/ (accessed on 11 April 2024)). We performed conserved domain prediction using the online tool Batch CD-Search available at https://www.ncbi.nlm.nih.gov/Structure/bwrpsb/bwrpsb.cgi (accessed on 11 April 2024). Subsequently, we utilized TBtools for comprehensive gene structure analysis and generated high-quality images depicting the results.

### 4.3. Promoter Cis-Acting Element Analysis of the ZmAAP1 Genes in Maize

To investigate the characteristics of cis-acting elements in the ZmAAP1 promoter region, we first utilized TBtools to extract 2000 base-pair upstream sequences of the promoters from the ZmAAP1 coding sequences of multiple maize inbred lines. Subsequently, these sequences were uploaded to the Plant Cis-Acting Regulatory Element (CARE) website [45] (http://bioinformatics.psb.ugent.be/webtools/plantcare/html/ (accessed on 26 August 2024) for predictive analysis to identify all cis-acting elements. Finally, we again utilized TBtools to collate and analyze the prediction results and generated corresponding graphical representations.

### 4.4. Public RNA-Seq Expression Data Analysis

Public RNA-seq data were retrieved from the Plant Public RNA-seq Database (http://ipf.sustech.edu.cn/pub/plantrna/ (accessed on 5 September 2024)), specifically the datasets PRJEB36014 [46], PRJNA520822 [47], and PRJNA226757 [48]. A heatmap of expression levels across tissues/stages was generated using TBtools, with clustering based on the Pearson correlation distance and the complete linkage method.

### 4.5. Plant Materials and Treatments

Gongzhuling City, Jilin Province, China, lies in the humid temperate zone and exhibits a prominent continental monsoon climate. The four seasons are distinct, with cold winters and hot summers. The annual average temperature is 5.6 °C, the annual average precipitation is 594.8 mm, and the frost-free period is 144 days. Significant seasonal variations were observed in temperature, rainfall, and sunlight. Spring is dry with frequent strong winds, but warming is rapid; summer is hot and rainy; autumn is warm, with many sunny days; and winter is long and cold. The distribution of precipitation throughout the year is uneven, with the highest precipitation in summer and the lowest precipitation in winter.

The experiment adopted a two-factor segmentation graph design. One factor was the genetic background of corn varieties, identified at ten different levels using labels such as A1, A2, A3, etc. Another factor was the application rate of nitrogen fertilizer. Fertilization during the three-leaf stage, with a total nitrogen gradient of 57 kg per 1000 m^2^ (high nitrogen 100% N), medium nitrogen treatment at 70% of the total nitrogen gradient application rate (medium nitrogen 70% N), and low nitrogen treatment at 0% (low nitrogen 0% N). The row spacing was 0.6 m, the length was 5.0 m, the plant spacing was 25 cm, and the planting density was 58,000 plants per hectare. Each treatment combination (genotype × nitrogen level) consisted of 3 biological replicate field plots (independent experimental units), with 3 technical replicates (sub-samples) collected from each plot to assess within-plot variability. Except for the variation in nitrogen fertilizer application rates, all other agronomic management practices (e.g., tillage, irrigation, and pest control) were standardized to align with commercial field production standards.

The performance of genetically modified maize varieties with high nutrient utilization efficiency was evaluated under three nitrogen application treatments: high nitrogen (100% N), medium nitrogen (70% N), and low nitrogen (0% N). The evaluation indicators included relative chlorophyll content (SPAD value) at different growth stages, plant height, spike length, spike diameter, the number of rows per spike, the number of grains per row, spike weight, hundred grain weight, endosperm length, grain yield, aboveground biomass yield, and total nitrogen content at plant maturity.

### 4.6. Cloning and Transformation of AtAAP1 and the Acquisition of Genetically Modified Maize

To study the function of the high-yield amino acid absorption and transport gene AtAAP1, the Arabidopsis AtAAP1 gene (At1g58360) was cloned into the expression vector pCAM-UPN containing the selectable marker, which generated the recombinant vector pCAM-UPN::AtAAP1 (Figure S5). This plasmid contains an independent T-DNA region with a total length of 5574 p. pCAM-UPN: The complete insertion fragment of the AtAAP1 vector contains two expression units—the target gene expression unit and the test marker gene expression unit. Among them, the expression unit of the target gene comprises the corn ubiquitin promoter (containing the ubiquitin region 5′UTR and the first intron) pZmUbi, the AtAAP1 sequence of the target gene, and the untranslated 3′(tNos) region of the coding sequence for the cochienic synthesis of Agrobacterium tumefaciens. The gene expression units of the screening marker comprise the 35S promoter of the flower giraffe mosaicovirus (CaMV) pCaMV35S, the gene bar of the screening marker encoding glyphosate acetyltransferase PAT, and the 35S terminator of the flower giraffe mosaicovirus (CaMV) tCaMV35S polyA. In addition, there are recognition sequences of the left boundary LB and recognition sequences of the right boundary RB, as well as non-coding skeletal sequences in the DNA-T zone of the vector.

Then, the recombinant vector carrying the cassette gene and the AtAAP1 expression band was introduced into the corn receptor material (HiII, a receptor maize inbred line) for agrobacterial transformation, giving rise to transformed plants [49]. After preliminary screening with herbicide glufosinate (corresponding to the stripe marker) and PCR verification of AtAAP1 integration, three positive transgenic plants were selected from 218 transformation events. The phenotypic analysis showed that these three transformants not only exhibited resistance to glufosinate but also significantly improved the absorption and transport efficiency of amino acids compared to wild plants.

### 4.7. Expression Analysis of ZmAAP1 in Different Development Stages and Different Tissues

Total RNA was extracted from different generations (T4, T5, and T6) of maize transformant plants subjected to different nitrogen treatments (HN, MN, and LN) and harvested at various growth stages (seedling stage, jointing stage, tasseling stage, and maturity stage). The RNA was extracted from different parts of the plants, including grains, roots, leaves, male spikes, stems, endosperms, and bracts, using a total RNA extraction kit provided by Tiangen Company. After ensuring the purity, concentration, and integrity of the extracted RNA, it was treated with the DNAase enzyme, followed by cDNA synthesis using reverse transcriptase. The cDNA was analyzed using qualitative PCR methods. ZmACTIN was used as an internal control, and the relative gene expression level was detected and calculated using the CT method (2^−ΔΔCt^) to examine the expression level of the target gene. GraphPad Prism 9 was utilized to generate graphs and perform statistical analysis to determine significant differences.

The monoclonal antibodies used in this experiment were provided by Shanghai Youlong Biotechnology Co., Ltd. The content of AtAAP1 protein in the roots, stems, leaves, silks, tassels, husks, and grains of transgenic maize plants in the intermediate experimental field was detected at the seedling, jointing, tasseling, and maturing stages. The specific steps followed are as follows:

Standard protein was diluted to solutions with concentrations of 1 ng/μL, 0.5 ng/μL, 0.25 ng/μL, 0.125 ng/μL, 0.0625 ng/μL, and 0.03125 ng/μL. After completely grinding 0.1 g of the sample, 1 mL of buffer was added, followed by centrifugation at 4000 rpm for 3 min. Then, 100 μL of the supernatant was added to a microplate, gently vortexed and mixed, and incubated at 25 °C in a light-protected environment for 45 min. The liquid in each well was aspirated, and each well was washed 4–5 times with 250 μL of washing buffer (with a 10 s interval between each wash) and then blotted dry with absorbent paper. Next, 100 μL of enzyme-labeled working solution was added to each well, gently vortexed and mixed, and incubated at 25 °C in a light-protected environment for 30 min. The plate was thoroughly washed 4–5 times with washing buffer. Subsequently, 100 μL of chromogenic agent was added to each well, followed by incubation at 25 °C in a light-protected environment for 15 min. Finally, 100 μL of stopping solution was added to each well and gently vortexed and mixed, and the optical density (OD) value of each well was measured at 450 nm and 630 nm. The content of the AtAAP1 protein was calculated based on these values.

## Figures and Tables

**Table 1:** Characteristics of AAP1 proteins.

Line	Gene ID	Chromosome	Gene Localization	Exon Number	Protein Length (aa)	Protein MW (da)	pI	Predicted Subcellular Localization
Arabidopsis	AT1G58360	Chr1	21,676,388..21,680,519	6	485	52,895.58	8.03	plas: 11, golg: 2
A188	Zm00056aa035386	Chr7	4,587,814..4,590,133	5	489	52,762.31	7.52	plas: 6.5, E.R.: 6, cyto_plas: 4
A632	Zm00092aa030742	Chr7	4,003,500..4,005,772	5	483	52,112.5	7.36	plas: 7.5, E.R.: 5, cyto_plas: 4.5
B104	Zm00007a00026974	scaffold7.87	124,806..126,646	5	512	55,367.24	8.10	cyto: 4, plas: 4, chlo: 2, pero: 2, E.R.: 1
B73V5	Zm00001eb299500	Chr7	4,131,802..4,134,061	5	483	52,122.54	7.34	plas: 6, E.R.: 6, chlo: 1
B84	ZmB84.07G013300	Chr7	4,176,235..4,178,492	5	483	52,122.54	7.34	plas: 6, E.R.: 6, chlo: 1
B97	Zm00018ab313690	Chr7	4,024,415..4,026,800	5	483	52,122.54	7.34	plas: 6, E.R.: 6, chlo: 1
Chang7-2	Zm00093aa028483	Chr7	4,434,277..4,436,571	5	484	52,252.69	7.52	plas: 7.5, E.R.: 6, cyto_plas: 4.5
CML103	Zm00021ab390530	Chr9	79,162,556..79,173,388	5	486	53,005.46	8.42	plas: 11, golg: 2
CML228	Zm00022ab304680	Chr7	4,189,276..4,191,617	5	483	52,096.46	7.34	plas: 7, E.R.: 5, chlo: 1
CML247	Zm00023ab307940	Chr7	3,970,939..3,973,184	5	483	52,110.48	7.34	plas: 6.5, E.R.: 5, cyto_plas: 4, chlo: 1
CML277	Zm00024ab305030	Chr7	4,042,444..4,044,744	5	482	52,069.43	7.34	plas: 6, E.R.: 6, chlo: 1
CML322	Zm00025ab310550	Chr7	4,116,561..4,118,973	5	483	52,110.48	7.34	plas: 6.5, E.R.: 5, cyto_plas: 4, chlo: 1
CML333	Zm00026ab302670	Chr7	4,553,074..4,555,471	5	483	52,152.56	7.34	plas: 6, E.R.: 6, chlo: 1
CML52	Zm00019ab287350	Chr7	4,330,757..4,333,005	5	483	52,122.54	7.34	plas: 6, E.R.: 6, chlo: 1
CML69	Zm00020ab389900	Chr9	81,667,572..81,678,365	5	486	52,991.39	7.97	plas: 11, golg: 2
Dan340	Zm00094aa029166	Chr7	4,162,675..4,164,968	5	483	52,152.56	7.34	plas: 6, E.R.: 6, chlo: 1
DK105	Zm00016a031965	Chr7	4,545,926..4,547,766	5	483	52,122.54	7.34	plas: 6, E.R.: 6, chlo: 1
EP1	Zm00010a028426	Chr7	4,773,048..4,774,862	5	483	52,110.48	7.34	plas: 6.5, E.R.: 5, cyto_plas: 4, chlo: 1
F7	Zm00011a028590	Chr7	4,727,434..4,729,290	5	483	52,096.46	7.34	plas: 6.5, E.R.: 5, cyto_plas: 4, chlo: 1
HP301	Zm00027ab304530	Chr7	4,245,097..4,247,332	5	483	52,110.48	7.34	plas: 6.5, E.R.: 5, cyto_plas: 4, chlo: 1
Huangzaosi	Zm00095aa029908	Chr7	5,054,785..5,057,125	5	483	52,152.56	7.34	plas: 6, E.R.: 6, chlo: 1
II14H	Zm00028ab307090	Chr7	4,142,656..4,145,113	5	483	52,069.43	7.34	plas: 6.5, E.R.: 5, cyto_plas: 4, chlo: 1
Jing-724	Zm00096aa031407	Chr7	4,151,056..4,153,290	5	483	52,122.54	7.34	plas: 6, E.R.: 6, chlo: 1
Jing-92	Zm00097aa030840	Chr7	4,101,721..4,103,880	5	483	52,108.51	7.34	plas: 6, E.R.: 6, chlo: 1
K0326Y	Zm00054a026320	Chr7	4,263,322..4,265,541	5	489	52,764.29	7.52	plas: 6.5, E.R.: 6, cyto_plas: 4
Ki11	Zm00030ab300360	Chr7	4,021,247..4,023,521	5	483	52,104.5	7.34	plas: 7, E.R.: 6
Ki3	Zm00029ab313690	Chr7	4,096,813..4,099,330	5	483	52,069.43	7.34	plas: 6.5, E.R.: 5, cyto_plas: 4, chlo: 1
Ky21	Zm00031ab311220	Chr7	4,452,423..4,454,691	5	484	52,240.64	7.52	plas: 6.5, E.R.: 6, cyto_plas: 4
la453	Zm00045a034145	Chr7	4,294,850..4,297,157	5	491	53,042.66	7.39	plas: 6.5, E.R.: 6, cyto_plas: 4
LH145	07G011200	Chr7	3,634,265..3,636,507	5	483	52,110.48	7.34	plas: 6.5, E.R.: 5, cyto_plas: 4, chlo: 1
LH244	Zm00052a033964	Chr7	4,185,287..4,187,577	5	483	52,110.48	7.34	plas: 6.5, E.R.: 5, cyto_plas: 4, chlo: 1
M162W	Zm00033ab317210	Chr7	4,442,058..4,444,280	5	483	52,138.54	7.34	plas: 7, E.R.: 5, chlo: 1
M37W	Zm00032ab312130	Chr7	4,501,878..4,504,131	5	483	52,110.48	7.34	plas: 6.5, E.R.: 5, cyto_plas: 4, chlo: 1
MEX	ZMex07t023391	Chr7	1,738,993..1,740,854	5	475	51,358.6	7.34	plas: 6, E.R.: 6, chlo: 1
Mo17	Zm00014ba322500	Chr7	4,062,253..4,064,583	5	483	52,122.54	7.34	plas: 6, E.R.: 6, chlo: 1
Mo18w	Zm00034ab320170	Chr7	4,257,931..4,260,225	5	483	52,110.48	7.34	plas: 6.5, E.R.: 5, cyto_plas: 4, chlo: 1
Ms71	Zm00035ab310900	Chr7	4,280,209..4,282,542	5	483	52,122.54	7.34	plas: 6, E.R.: 6, chlo: 1
NC350	Zm00036ab308740	Chr7	4,224,252..4,226,570	5	483	52,096.46	7.34	plas: 7, E.R.: 5, chlo: 1
NC358	Zm00037ab303850	Chr7	4,311,993..4,314,286	5	483	52,168.52	7.34	plas: 6.5, E.R.: 5, cyto_plas: 4, chlo: 1
NKH8431	07G011700	Chr7	4,167,721..4,169,992	5	483	52,112.5	7.36	plas: 7.5, E.R.: 5, cyto_plas: 4.5
Oh43	Zm00039ab305460	Chr7	4,154,135..4,156,404	5	483	52,108.51	7.34	plas: 6, E.R.: 6, chlo: 1
Oh7B	Zm00038ab306300	Chr7	4,347,306..4,349,531	5	483	52,122.54	7.34	plas: 6, E.R.: 6, chlo: 1
P39	Zm00040ab318650	Chr7	4,106,064..4,108,397	5	483	52,122.54	7.34	plas: 6, E.R.: 6, chlo: 1
PE0075	Zm00017a032359	Chr7	4,602,676..4,604,512	5	483	52,108.51	7.34	plas: 6, E.R.: 6, chlo: 1
PH207	Zm00008a034417	Chr9	78,976,202..78,988,500	6	486	53,064.49	8.42	plas: 11, golg: 2
PHB47	07G012600	Chr7	3,469,964..3,472,236	5	483	52,110.48	7.34	plas: 6.5, E.R.: 5, cyto_plas: 4, chlo: 1
PHJ40	07G009200	Chr7	2,969,776..2,972,048	5	483	52,170.54	7.34	plas: 6.5, E.R.: 5, cyto_plas: 4, chlo: 1
S37	Zm00100aa029860	Chr7	3,833,435..3,835,747	5	483	52,108.51	7.34	plas: 6, E.R.: 6, chlo: 1
SK	Zm00015a029128	Chr7	4,492,711..4,495,028	5	482	52,054.42	7.20	plas: 6, E.R.: 6, chlo: 1
Tx303	Zm00041ab311140	Chr7	4,491,349..4,493,647	5	484	52,226.61	7.52	plas: 6.5, E.R.: 6, cyto_plas: 4
Tzi8	Zm00042ab312990	Chr7	4,416,409..4,418,656	5	483	52,110.48	7.34	plas: 6.5, E.R.: 5, cyto_plas: 4, chlo: 1
W22	Zm00004b034751	Chr7	4,377,245..4,379,604	5	489	52,764.29	7.52	plas: 6.5, E.R.: 6, cyto_plas: 4
Xu178	Zm00101aa029717	Chr7	4,227,421..4,229,786	5	483	52,122.54	7.34	plas: 6, E.R.: 6, chlo: 1
Ye478	Zm00102aa030247	Chr7	3,734,826..3,737,136	5	483	52,110.48	7.34	plas: 6.5, E.R.: 5, cyto_plas: 4, chlo: 1
Zheng58	Zm00103aa030073	Chr7	4,026,638..4,028,804	5	483	52,110.48	7.34	plas: 6.5, E.R.: 5, cyto_plas: 4, chlo: 1

**Table 2:** Information on maize lines participating in the test.

Variety Name	Female Parent	Male Parent
ZD958	Zheng58	Chang7-2
ZD958 (−/#)	Zheng58-AtAAP1-1-aa	Chang7-2
ZD958 (−/−)	Zheng58-AtAAP1-1-aa	Chang7-2-AtAAP1-1-aa
ZD958(+/#)	Zheng58-AtAAP1-1-AA	Chang7-2
ZD958(+/+)	Zheng58-AtAAP1-1-AA	Chang7-2-AtAAP1-1-AA

Table note: − denotes a negative transgenic line; + denotes a positive transgenic line; # denotes a non-transgenic inbred line.

## Data Availability

The data presented in this study are available on request from the corresponding author.

## Supplementary Materials

The following supporting information can be downloaded at: https://www.mdpi.com/article/10.3390/plants14142242/s1, Figure S1: Southern Blot Hybridization Results of the Target Gene AtAAP1. Note: M: DNA Molecular-Weight Marker II DIG-labeled; 1-3: Sac I enzyme-digested T6-T4 generation HiII-AtAAP1-1; 4: Sac I enzyme-digested negative control HiII; 5-6: Sac I, Hind III enzyme-digested positive plasmid (pCAM-UPN::AtAAP1 plasmid); 7: Hind III enzyme-digested negative control HiII; 8-10: Hind III enzyme-digested T4-T6 generation HiII-AtAAP1-1. Figure S2: Southern Blot Hybridization Results of the Selection Marker Gene bar. Note: A. Hind III; B. Sac I; M: Trans 15K DNA Marker; 1: Positive plasmid (pCAM-UPN::AtAAP1 plasmid); 2: Negative control HiII; 3-5: T4-T6 generation HiII-AtAAP1-1. Figure S3: PCR detection of transgenic maize na1 across three consecutive generations. Note: A, C, E. AtAAP1; B, D, F. bar; A, B represents na1, C, D represents na2, E, F represents na3; M: DL 2000 Marker; 1: Water; 2: Negative control (HiII); 3: Positive control (pCAM-UPN::AtAAP1 plasmid); 4-6: T4-T6 generation. Figure S4: Phenotypic analysis of AtAAP1-transgenic maize under different nitrogen gradients. Note:(AAP1) indicates the maternal parent with the introduced AtAAP1, while (AAP1+AAP1) represents homozygous transgenic plants with both parental lines carrying the AtAAP1 gene. HN: high nitrogen; MN: medium nitrogen; LN: low nitrogen. Figure S5: Map of target genes and vector construction. The transformant HiII-AtAAP1 is obtained by loading the amino acid permease gene AtAAP1 into the target vector pCAM UPN (with bar gene), resulting in pCAM UPN: AtAAP1. It is then transferred into the recipient maize HiII through Agrobacterium mediated method and further screened, identified, and cultivated to obtain it. PCAM-UPN: The AtAAP1 plasmid contains an independent T-DNA region with a total length of 5574 bp.
