# Supplementary material for: Genetic Improvement and Functional Characterization of AAP1 Gene for Enhancing Nitrogen Use Efficiency in Maize
Source: Plants (Basel). 2025 Jul 21;14(14):2242. doi: 10.3390/plants14142242 (PMC12298070; doi:10.3390/plants14142242)
Supplement: Supplementary file 1 [file plants-14-02242-s001.zip › plants-3710585-supplementary.pdf]

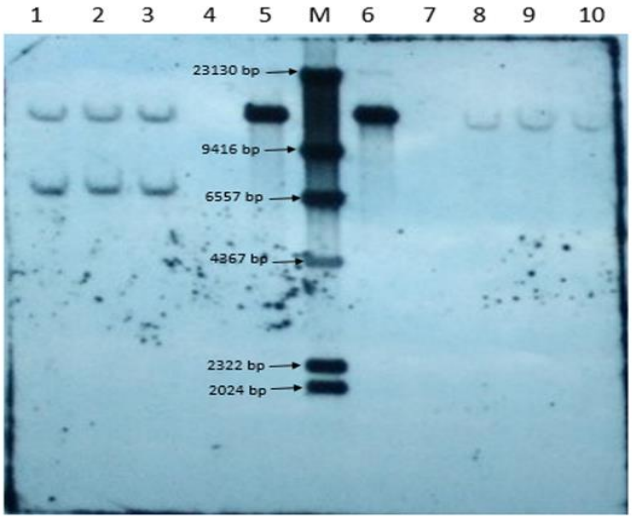

Figure S1. Southern Blot Hybridization Results of the Target Gene AtAAP1. Note: M: DNA Molecular-Weight Marker II DIG-labeled; 1-3: Sac I enzyme-digested T6-T4 generation HiII-AtAAP1-1; 4: Sac I enzyme-digested negative control HiII; 5-6: Sac I、Hind III enzyme-digested positive plasmid (pCAM-UPN::AtAAP1 plasmid); 7: Hind III enzyme-digested negative control HiII; 8-10: Hind III enzyme-digested T4-T6 generation HiII-AtAAP1-1.

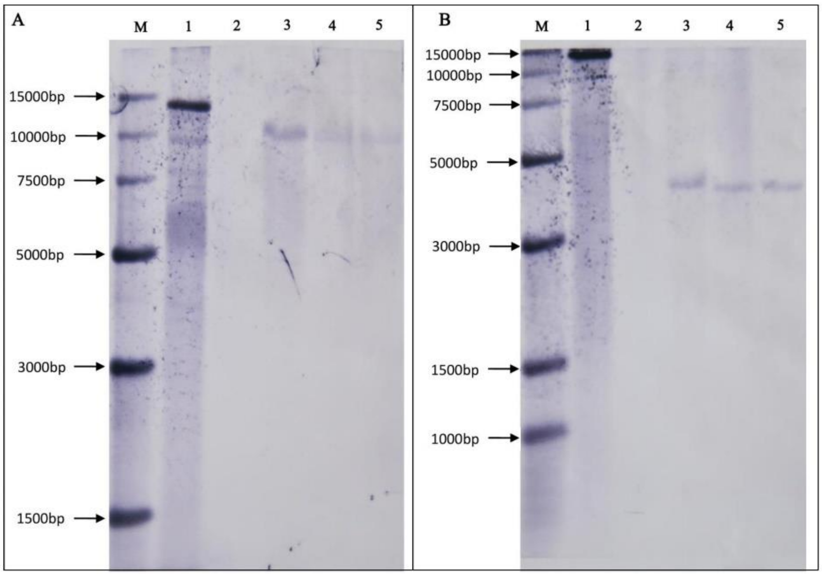

Figure S2. Southern Blot Hybridization Results of the Selection Marker Gene bar. Note: A. Hind III; B. Sac I; M: Trans 15K DNA Marker; 1: Positive plasmid (pCAM-UPN::AtAAP1 plasmid); 2: Negative control HiII; 3-5: T4-T6 generation HiII-AtAAP1-1.

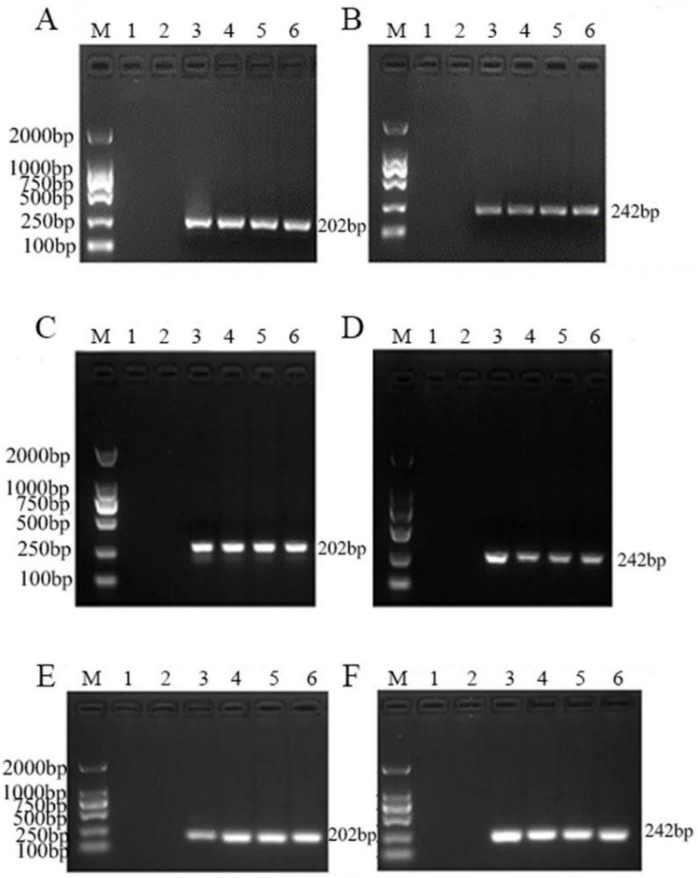

Figure S3. PCR detection of transgenic maize na1 across three consecutive generations. Note: A、C、E. AtAAP1; B、D、F. bar; A、B represents na1, C、D represents na2, E、F represents na3; M: DL 2,000 Marker; 1: Water; 2: Negative control (HiII); 3: Positive control (pCAM-UPN::AtAAP1 plasmid); 4-6: T4-T6 generation.

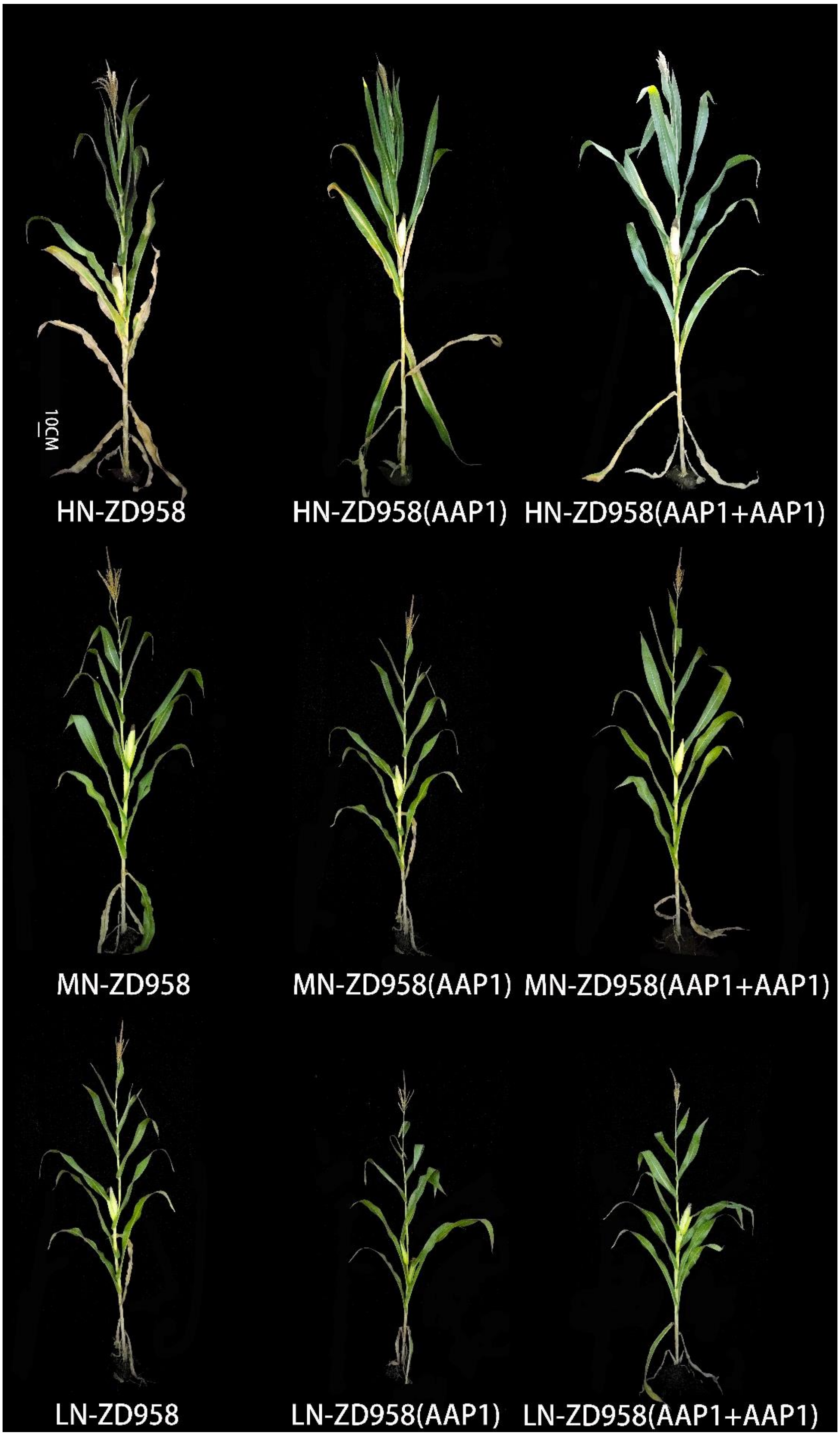

Figure S4. Phenotypic analysis of AtAAP1-transgenic maize under different nitrogen gradients. Note:(AAP1) indicates the maternal parent with the introduced AtAAP1, while (AAP1+AAP1) represents homozygous transgenic plants with both parental lines carrying the AtAAP1 gene. HN: high nitrogen; MN: medium nitrogen; LN: low nitrogen.

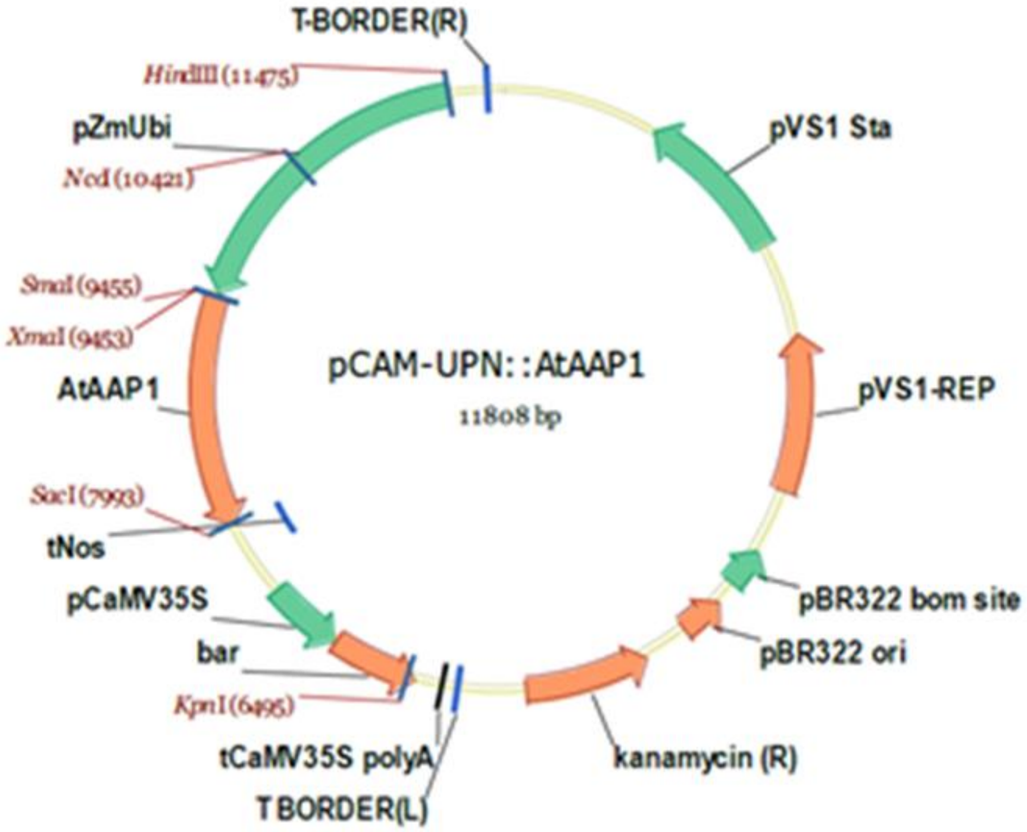

Figure S5. Map of target genes and vector construction. The transformant HiII-AtAAP1 is obtained by loading the amino acid permease gene AtAAP1 into the target vector pCAM UPN (with bar gene), resulting in pCAM UPN: AtAAP1. It is then transferred into the recipient maize HiII through Agrobacterium mediated method and further screened, identified, and cultivated to obtain it. PCAM-UPN: The AtAAP1 plasmid contains an independent T-DNA region with a total length of 5574 bp.
